# A novel telerehabilitation with an educational program for caregivers using telelecture is feasible for fall prevention in elderly people: A case series

**DOI:** 10.1097/MD.0000000000027451

**Published:** 2022-02-11

**Authors:** Kentaro Moriichi, Mikihiro Fujiya, Takanori Ro, Tetsuo Ota, Hitomi Nishimiya, Mariko Kodama, Nana Yoshida, Yukari Hattori, Tetsuya Hosokawa, Hohei Hishiyama, Masao Kunimoto, Hiroki Hayashi, Hiroyuki Hirokawa, Akitoshi Yoshida

**Affiliations:** a Joint Research Department of Telemedicine and Telecare, Asahikawa Medical University, Asahikawa, Japan; b Gastroenterology and Endoscopy, Division of Metabolism and Biosystemic Science, Gastroenterology, and Hematology/Oncology, Department of Medicine, Asahikawa Medical University, Asahikawa, Japan; c Rehabilitation Unit, Asahikawa Medical University Hospital, Asahikawa, Japan; d Department of Physical Medicine and Rehabilitation, Asahikawa Medical University Hospital, Asahikawa, Japan; e Department of Nursing, Asahikawa Medical University, Asahikawa, Japan; f Chuwa Clinic, Asahikawa, Japan; g Kunimoto Hospital, Asahikawa, Japan; h Telemedicine Center, Asahikawa Medical University, Asahikawa, Japan; i Management Planning Department, Asahikawa Medical University Hospital, Asahikawa, Japan; j Asahikawa Medical University, Asahikawa, Japan.

**Keywords:** case series, falls, physical activity, rehabilitation, telemedicine

## Abstract

**Background:**

The importance of fall prevention rehabilitations has been well recognized. Recently telerehabilitation was developed, however, there have been no reports on telerehabilitation with direct support from specialists for fall prevention among the elderly. We herein reported telerehabilitation by caregivers educated by our novel educational program.

**Methods:**

Caregivers were educated with our educational program using a telelecture system and supported telerehabilitation following instructions from rehabilitation specialists in our university using the telemedicine system every two to four weeks for three months. Caregivers were assessed with our original questionnaire before and after the telelecture. Participants were assessed by the Berg Balance Scale (BBS), Timed Up & Go test (TUG test), Hand-held dynamometer (HHD) and Mini-Mental State Examination (MMSE) before and after telerehabilitation. Wilcoxon's signed-rank test was used for the statistical analyses. A value of *P*<.05 was considered statistically significant.

**Results:**

Nine elderly people were enrolled. The mean age was 84.7 (78–90) years old and the sex ratio was 1:8 (males:females). The average number of telerehabilitation sessions was 4.7. The average score of nineteen caregivers before the lecture was 15.3, while that after the lecture was 18.3. Caregivers’ understanding was significantly increased after the telelecture (*P*<.001). No adverse events occurred during the study period. The median values of the BBSs, TUG test, right and left HHD and MMSE before and after 3 months’ telerehabilitation were 43 (95% confidence interval [CI]: 40.10, 49.01) and 49 (95% CI: 41.75, 50.91), 17.89 (95% CI: 15.51, 23.66) and 18.53 (95% CI: 14.56, 25.67), 7.95 (95% CI: 4.38, 10.14) and 11.55 (95% CI: 7.06, 13.55), 9.85 (95% CI: 6.79, 12.59) and 13.20 (95% CI: 7.96, 14.42), and 19 (95% CI: 12.34, 21.66) and 16 (95% CI: 10.81, 21.00), respectively. Although approximately half of the participants showed improvement in the BBS, TUG test, right and left HHD and MMSE, no significant changes were observed (*P*=.7239, *P*=.3446, *P*=.1023, *P*=.3538 and *P*=.8253, respectively).

**Conclusions:**

Our telerehabilitation program exhibited significant effects in elderly people and improved the degree of understanding concerning rehabilitation among caregivers in facilities for elderly people.

## 1 Introduction

It is estimated that approximately 30% of community dwelling people of ≥65 years of age experience a fall at least once per year.^[1]^ Residents of institutions showed a higher rate of fall in comparison to elderly people living in houses.^[2]^ In Japan, Yasumura et al reported that the prevalence of falls was 12.3–26.9%.^[3]^ Falls, as well as pneumonia, are events that can threaten the health status of elderly adults, and which have the potential to adversely impact their activities of daily living. Indeed, falls account for 19.3% of deaths in Japan.^[3]^ According to some studies that investigated the risk factors for fall in elderly people,^[4]^ muscle weakness, balance and gait deficits are the main causes of fall. It is therefore crucial to prevent falls among the elderly and rehabilitation for fall prevention should be widely performed because few elderly people recover their muscle strength and body balance without rehabilitation.

Although various rehabilitation strategies have been applied in the clinical setting for a long time, most require face-to-face therapy. Thus, the patients who want to receive rehabilitation have to travel to rehabilitation centers or hospitals, which involves time, financial costs^[5]^ and increases risk of COVID-19 infection. In addition, nursing staff in rural areas cannot easily obtain the latest knowledge on rehabilitation because it can be difficult to attend medical lectures or meetings.^[6]^ With regard to rehabilitation, medical inequality between rural and urban populations have been an object of discussion for many years.^[7–10]^ Wakerman and Humphreys reported the health disadvantage of rural residents in Australia and noted that they experienced poorer health outcomes in comparison to urban residents.^[11]^ In line with these findings, Bradford et al. showed that the mortality and life expectancy of people living in rural areas were poorer in comparison to people living in urban areas.^[12]^

Recently new telecommunication-based methods that enable the patients to receive rehabilitation in their own home have been developed and applied in the field of rehabilitation.^[13]^ While telerehabilitation has been clinically applied, most patients have been middle-aged adults with diseases including multiple sclerosis,^[14]^ stroke^[15]^ and cardiac disease.^[16]^ Thus, the efficacy of telerehabilitation for fall-prevention in elderly people has been totally unclear. In this pilot study, we proposed a novel procedure for fall-preventive telerehabilitation for elderly people, which combined a physical therapist (PT)-assisted and personalized telerehabilitation program with a caregiver-education program.

We hypothesized that our telerehabilitation system had an equal effect to conventional rehabilitation. The establishment of our telemedicine system would offer an equal quality of rehabilitation to elderly persons irrespective of their location, leading to a reduction of time and financial costs for the medical staff, including nurses, caregivers and physical therapists and would reduce the risk of infection with infectious diseases, such as COVID-19. In addition, nursing staff would also have an opportunity to obtain the latest rehabilitation information through the system.

## 2 Methods

This study was approved by the institutional review board of Asahikawa Medical University (IRB # 17191). All participants provided written, informed consent. Consent for publication was obtained from all participants.

### 2.1 Telemedicine systems

**Figure 1:**
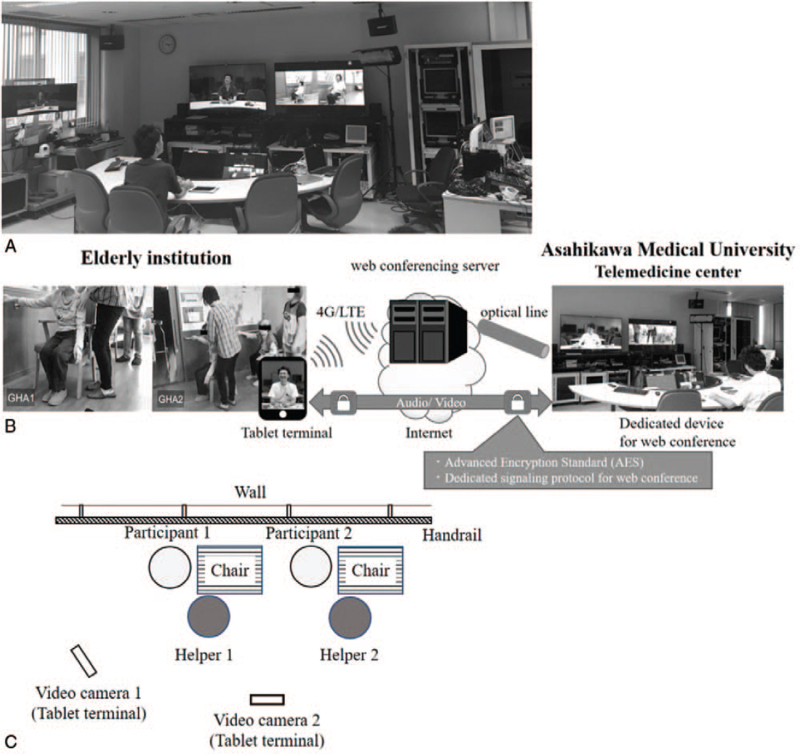
The Telemedicine center and a Bird's-eye view of telerehabilitation. A distant view of the Telemedicine center in Asahikawa Medical University; the lecturer sits in front of the video camera and monitors. The operator monitors the telerehabilitation session (A). A schematic illustration of the telemedicine system. Each institution can connect to the telemedicine network via a broadband Internet connection (e.g., asymmetric digital subscriber line [ADSL] or fiberoptic connection using a tablet or laptop PC (B). Participants exercise along the wall with a handrail to prevent staggering and caregivers stand beside the participants. One video camera (tablet terminal) is placed diagonally to the participant; the other is placed by their side (C).

A telemedicine system was installed in the Telemedicine Center of Asahikawa Medical University (Fig. 1A). The VidyoRoom HD230 software program (Vidyo, Inc., Hackensack, USA) was used for telemedicine conferences, lectures and rehabilitation sessions in the telemedicine center. The VidyoMobile software program (Vidyo, Inc., Hackensack, USA) was used in the institutions, which included elderly group homes and nursing homes. Each institution can connect to the telemedicine network via a broadband Internet connection (e.g., an asymmetric digital subscriber line [ADSL] or fiberoptic line) using a tablet or laptop PC. The maximum image quality of the system is 1080pHD and the images are shrinked by H.264/Scalable Video Coding (SVC), an original signaling protocol developed by Vidyo, Inc. The security of our telemedicine system consists of Advanced Encryption Standard (AES) encryption and a Secure Sockets Layer (SSL) (Fig. 1B). Two tablet terminals are used as video cameras in each institution. To view all participants, one tablet terminal was placed diagonally to the participant and the other was placed by their side (Fig. 1C). Specialists, including medical doctors (MDs) from the rehabilitation department, PTs and nurses gave lectures to staff members in all institutions and created a personalized telerehabilitation plan for each patient.

### 2.2 Education for caregivers

**Figure 2:**
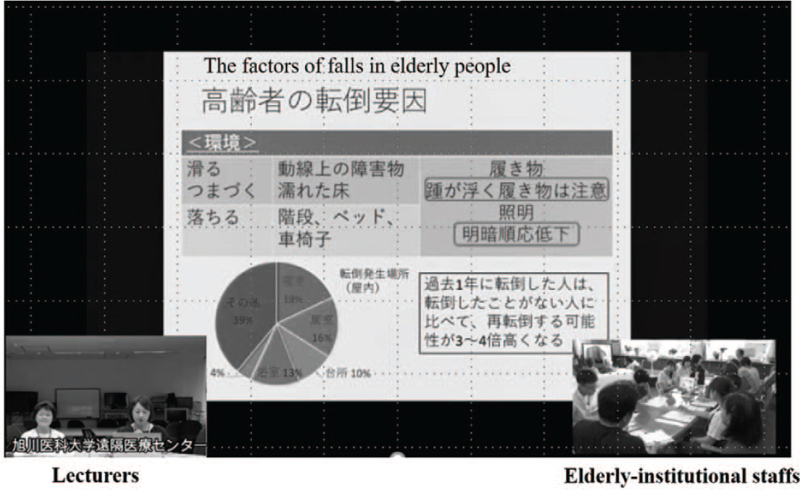
Education for caregivers. Lectures were given to staff members of elderly institutions using a telemedicine system and bidirectional discussions were held.

Before the telerehabilitation was started, medical staff (MDs, nurses and PTs) in Asahikawa Medical University gave lectures on rehabilitation to staff members at elderly institutions, including caregivers and nurses using the teleconference system. In these lectures, nurses talked about the mechanism of falling and fall prevention and PTs explained the actual methods of the rehabilitation using original materials based on the guidelines or consensus. To evaluate the efficacy of the lectures, 13 staff members from elderly institutions took the same test regarding knowledge in relation to falls before and after the lectures. The test contained 20 questions in eight categories: the relationship between falls and Living environments, bone fracture, medication use, a past history of falls, disease, the reasons for requiring nursing care, and dementia (Fig. 2).

### 2.3 Participant selection

#### 2.3.1 Inclusion criteria

The inclusion criteria were as follows:

1. clear consciousness;
2. stable respiratory and circulation status;
3. ability to communicate and exercise while following directions;
4. ability to walk with or without walk aids;
5. ability to exercise with a caregiver's assistance with or without knowledge about rehabilitation,
6. the provision of informed consent by the participant or their family.

Even when a participant met all of these conditions above, rehabilitation was cancelled when the following conditions were not met just before the rehabilitation session:

1. a resting heart rate of 40–120 beats/min;
2. a resting systolic blood pressure from 70–200 mmHg;
3. a resting diastolic blood pressure of <120 mmHg;
4. no effort angina pectoris;
5. no atrial fibrillation with remarkable bradycardia or tachycardia;
6. a stable circulation status without recent history of cardiac infarction;
7. no remarkable arrhythmia;
8. no chest pain at rest;
9. no palpitations, shortness of breath or chest pain;
10. no nystagmus, cold sweat or nausea;
11. body temperature <38°C;
12. blood oxygen saturation (SaO_2_) at rest >90%.

#### 2.3.2 Exclusion criteria

The following exclusion criteria were applied:

1. resting systolic blood pressure outside the range of 70–200 mmHg;
2. resting diastolic blood pressure ≥120 mmHg;
3. resting heart rate of ≤40 beats/min or ≥120 min;
4. poorly controlled arrhythmia;
5. ischemic heart disease such as acute myocardial infarction, unstable angina pectoris with symptoms of heart failure;
6. pulmonary disease with cyanosis and/or a SaO_2_ value of ≤ 90%;
7. poorly controlled diabetes mellitus (DM); DM with complications, including neuropathy, retinopathy and nephropathy.

#### 2.3.3 Questionnaire

**Figure 3:**
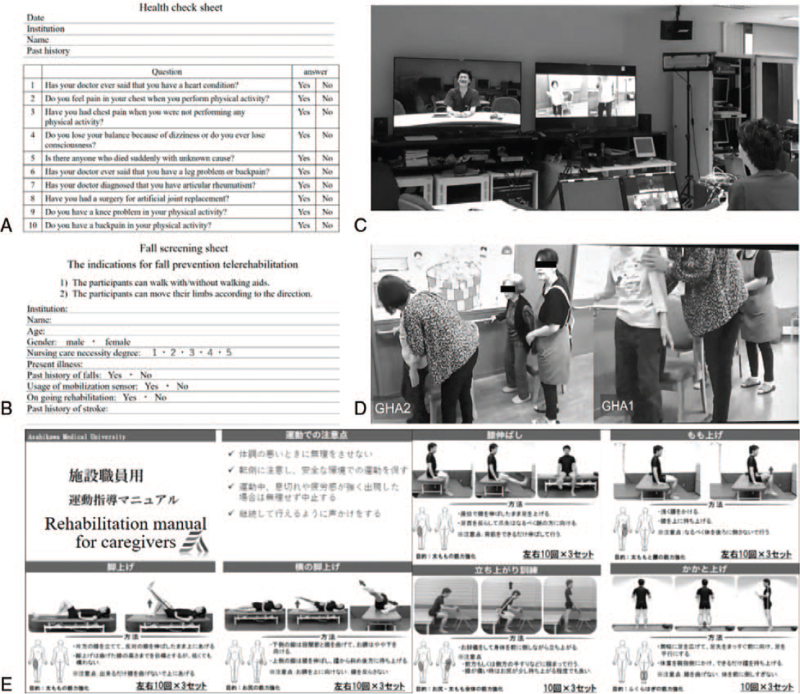
Questionnaires, telerehabilitation and the rehabilitation manual. Questionnaires on the participants’ health status (A) and a fall screening sheet (B). MDs, PTs and nurses in Asahikawa Medical University could decide the indications for telerehabilitation based on these questionnaires. Actual scenes of telerehabilitation (C&D) and the rehabilitation manual (E). A PT asked participants about their health condition and directed exercise via the telemedicine system. MDs and PTs proposed a personalized rehabilitation menu based on the rehabilitation manual.

We modified the Physical Activity Readiness Questionnaire (PAR-Q) to assess the health status of the telerehabilitation participants. The participants or their family members answered the questions. The questionnaire consists of ten questions related to the patient's health history, current symptoms, and risk factors to determine the safety of rehabilitation and possible risks associated with their participation (Fig. 3A).

#### 2.3.4 Fall screening sheet

To evaluate the indications for rehabilitation, the participants’ information was searched by nursing staff using the fall screening sheet. The fall screening sheet contained information about the participants, including their age, present illness, past history of falls and stroke, nursing care levels, usage of fall prevention alarm systems, and rehabilitation experience (Fig. 3B)

#### 2.3.5 Selection of participants

Based on the above information, MDs, PTs and nurses in Asahikawa Medical University discussed the selection of participants with staff members of elderly institutions using telemedicine systems, then the telerehabilitation participants were selected. All selected elderly people participated in the telerehabilitation program.

### 2.4 Telerehabilitation programs

MDs, nurses and PTs selected a personalized program for each participant from the following rehabilitation menus based on the “Questionnaire” and “Fall screening sheet” as well as the physical and mental condition of each participant using the telemedicine system. The rehabilitation menus contained six exercises (rising training, high knee training, straight leg raise training, side lying leg lift training, heel raise training and a knee straightening exercise), which mainly focused on muscular strength training of the lower limbs and the improvement of sitting balance. At the first session, the rehabilitation session was observed by MDs, nurses and PTs using the telerehabilitation system. The participants were required to continue the rehabilitation two or three times per week by themselves with caregiver's support in their nursing home. The interval between rehabilitation sessions depended on the participants’ rehabilitation program and condition. All participants underwent telerehabilitation five times in three months (first time, one week, one month, two months and three months later, respectively). (Fig. 3C–E)

### 2.5 Points of evaluation

A nurse visited elderly institutions and evaluated the efficacy of telerehabilitation for each participant using quantitative scales, including muscle strength, the Berg Balance Scale (BBS),^[17]^ the Timed Up & Go test (TUG test)^[18]^ and the Mini-Mental State Examination (MMSE)^[19]^ before the rehabilitation, and after one, two, and three months of rehabilitation. Two individuals (HN and TH) from our department collected, anonymized and preserved the data in on a password-protected PC. Thereafter they administered the data.

#### 2.5.1 Muscle strength

Our rehabilitation system is intended for use in rural areas where the number of medical specialists is quite limited. However, it is difficult for non-medical specialists, such as caregivers to measure the strength of all of the targeted muscles using a handheld dynamometer (HHD; μTas F-1, Anima Corp., Tokyo).^[20]^ The HHD showed a high intraclass correlation coefficient (ICC: 0.94–0.96) and its reliability and validity were proven.^[20]^ Among these muscles, the strength of the quadriceps femoris muscle was the easiest to measure with the HHD. Thus, we only measured the knee extensor muscle strength using the HHD. Briefly, with the participant sitting on a chair, the HHD sensor is attached to the limb with a belt that is anchored to a fixed structure. The participant extends the limb, and then knee extensor muscle strength is measured using the HHD.

#### 2.5.2 Berg balance scale

To evaluate participants’ balance ability, all participants were assessed by the BBS.^[17]^ The BBS is thought to be reliable and widely accepted method for the evaluation of balance.^[21]^ Downs et al reported that the relative inter-rater and intra-rate reliability of the BBS was 0.98 and 0.97, respectively.^[21]^ Briefly, this scale contains 14 items related to sitting balance, standing balance and dynamic balance. The scores for each item range from 0 to 4, with a score of 0 representing inability to complete the task and a score of 4 representing independent completion of the task.

#### 2.5.3 Timed up & go test

The TUG test has been widely used to evaluate basic mobility maneuvers that are frequently performed by the elderly population.^[18,22]^ Podsiadlo et al evaluated TUG test times in 60 patients and reported high inter-rater and intra-rater reliability, and high correlation with the BBS, suggesting that the TUG time appeared to predict a patient's ability to walk safely.^[18]^ The TUG test measures the time that it takes for a person to rise from a chair, walk three meters, turn around, walk back to the chair, and sit down.^[18]^

#### 2.5.4 Mini-Mental state examination (MMSE)

The MMSE was developed to measure cognitive impairment in 1975^[19]^ and is currently used for dementia screening. The MMSE showed not only good test-retest reliability (0.80–0.95)^[23]^ but also acceptable sensitivity and specificity for the detection of mild to moderate stages of dementia.^[23]^ The MMSE is a 30-point questionnaire, with five sections (orientation [10 points], registration [3 points], attention and calculation [5 points], recall [3 points] and language [9 points]).^[24]^ The cut-off score for the diagnosis of dementia is reported to be <24, which is considered to be a standard criterion.^[25,26]^

### 2.6 Statistical analyses

Wilcoxon's signed-rank test was used for the statistical analyses of the pre and post test regarding knowledge in relation to falls and the pre-post evaluation of telerehabilitation, including the BBS, TUG test, HHD and MMSE. A value of *P*<.05 was considered statistically significant.

## 3 Results

### 3.1 Participants

Nine elderly people were selected from 24 people receiving care in elderly institutions using the above-described selection form. All participants were living in nursing homes. The mean age of the participants was 84.6 ± 4.5 years. One participant was male; 8 were female. Of the nine participants, two participants used walking aids and four participants had past history of fall on at least one occasion (Table 1). Written informed consent was obtained from all of the participants. There were no patients who dropped out of the rehabilitation program.

**Table 1:** The characteristics of participants and basic scores before the telerehabilitation.

					Pre rehabilitation				
Case	Age rang Gender	Walking aids	History of falls	Present	BBS	TUG (sec)	rt-HHD (kg)	lt-HHD (kg)	MMSE
1	70s/F	No	+	DM^∗^, HT^†^, Breast cancer p/o	51	15.73	7.95	9.65	25
2	90s/F	No	+	Dementia, DL^‡^, Gonitis, old Tbc	38	15.96	14.05	5.35	6
3	80s/F	Yes	−	Dementia, Collagen disease, Cataract	43	N/A	10.65	15.10	11
4	80s/F	No	−	Dementia, HT	43	27.74	4.05	6.80	22
5	70s/F	Yes	−	Dementia, CKD^§^	37	25.75	3.30	9.85	19
6	80s/F	No	−	Dementia	39	17.58	2.60	3.80	20
7	80s/F	No	+	Dementia, HT, femoral fracture	50	14.57	9.05	10.70	13
8	80s/F	No	−	Dementia, HT, Osteoporosis	49	18.20	5.65	13.35	19
9	80s/M	No	−	Dementia	51	21.16	8.05	12.60	8

∗ Diabetes mellitus.

† Hypertension.

‡ Dyslipidemia.

§ Chronic kidney isease.

### 3.2 Nursing staff education

**Figure 4:**
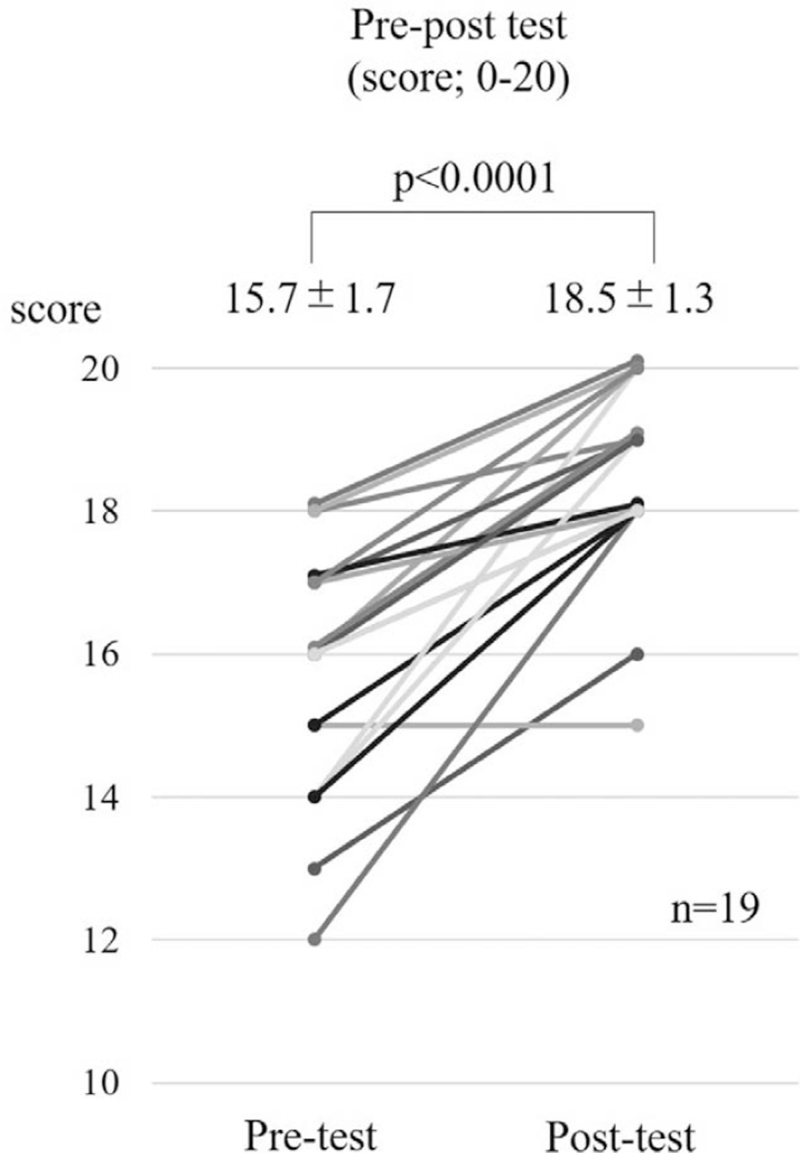
The efficacy of nursing staff education. The lecture significantly improved the total test scores of the nursing staff.

Nineteen staff members of elderly institutions including nurses and caregivers took the test. The average score before the lecture was 15.3, while that after the lecture was 18.3. The lecture significantly improved the total test score (*P* < .001). The category that showed the lowest score before the lecture was the reasons for requiring nursing care”, which showed significant improvement after the lecture, illustrating the efficacy of the telerehabilitation education program for staff members of elderly institutions (Fig. 4).

### 3.3 Berg balance scale

**Figure 5:**
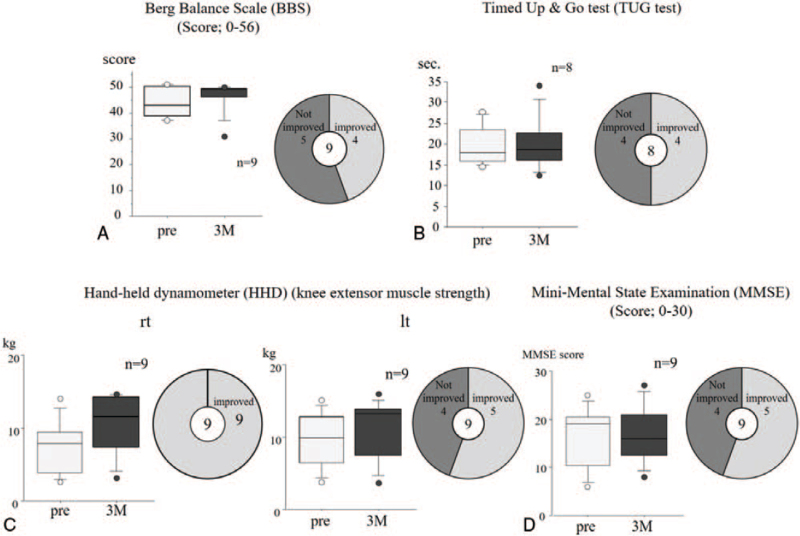
The changes in quantitative physical scales. The results of the Berg Balance Scale (BBS) (A), Timed Up & Go test (TUG test) (B), Hand-held dynamometer (HHD) (C) and Mini-Mental State Examination (MMSE) (D) values are shown. Approximately half of the participants showed improved BBS, TUG, or MMSE values after telerehabilitation. All participants showed improved right lower limb strength.

Four of the nine participants showed improved BBS values after telerehabilitation. The median BBS values before rehabilitation and after one, two, and three months of rehabilitation were 43 (95% CI: 40.10, 49.01), 44 (95% CI: 41.97, 48.48), 48 (95% CI: 42.48, 49.52) and 49 (95% CI: 41.75, 50.91) respectively, and increased with time (Fig. 5A). This suggests that the telerehabilitation is a feasible option for improving the body balance of elderly people.

### 3.4 Timed up & go test

Eight participants were assessed by TUG test twice while one participant declined to participate in the first TUG test due to anoesia. The median TUG times before and after the rehabilitation were 17.89 (95% CI: 15.51, 23.66) and 18.53 (95% CI: 14.56, 25.67), respectively, suggesting no effect of the rehabilitation. However, when the average TUG times before and after rehabilitation in each subject were compared, the time was shortened in 4 of the 8 participants, indicating that the telerehabilitation may improve the exercise ability for some elderly people (Fig. 5B).

### 3.5 Muscle strength

All participants showed improved right knee muscle strength and five of the nine participants showed improved left knee muscle strength. After rehabilitation, the median right and left knee extensor strength increased from 7.95 kg (95% CI: 4.38, 10.14) and 9.85 kg (95% CI: 6.79, 12.59) to 11.55 kg (95% CI: 7.06, 13.55) and 13.20 kg (95% CI: 7.96, 14.42), respectively, suggesting that the telerehabilitation improved the muscle strength of the lower limbs (Fig. 5C).

### 3.6 Mini-mental state examination (MMSE)

The median MMSE scores before and after 3 months of rehabilitation were 19 (95% CI: 12.34, 21.66) and 16 (95% CI: 10.81, 21.00), respectively. Despite the short study period, five of nine participants showed improvement (Fig. 5D), suggesting the usefulness of telerehabilitation for improving the cognitive status as well as physical activity.

### 3.7 Adverse events

All participants completed all procedures without any adverse events. No participants fell or showed any health problems during telerehabilitation and home exercise, suggesting the safety of the telerehabilitation program.

## 4 Discussion

This pilot study demonstrated the efficacy of the telerehabilitation for preventing falls in the elderly population. All participants could complete the telerehabilitation program, including the exercises, by themselves with support from caregivers. Around half of participants showed improved BBS or TUG values. It is noteworthy that muscle strength, particularly right knee extension muscle strength, improved in all participants. These results indicated that telerehabilitation showed potential for the prevention of falls in the elderly population.

Several studies have shown the efficacy of fall prevention rehabilitation for nursing home residents^[27,28]^ and community-dwelling older people.^[29–32]^ Community-based fall prevention interventions are low cost and low-tech programs, which can achieve a 25–30% reduction in falls per year after the program.^[33]^ However, interventions are often provided by non-medical specialists or less-integrated staff^[33]^ and face-to-face procedures are required, which would make these interventions difficult to implement in rural areas. To solve this problem, it is crucial to establish standardized telerehabilitation methods. While telecommunication-based rehabilitation methods have recently been developed,^[13]^ there have been no trials of telerehabilitation focused on preventive fall rehabilitation for general elderly people.^[33]^ Our results regarding the indices of muscle strength and balance showed no statistically significant improvement. Many studies on the efficacy of fall prevention rehabilitation or program showed the worsening of the functional indices such as gait speed, muscle strength and balance in non-intervention groups.^[34–36]^ Pérula et al reported that the average scores for gait and balance in a control group became significantly worse in comparison to baseline values during the study period.^[37]^ These suggest that in almost cases, elderly people cannot recover muscle strength and body balance without rehabilitation. While our case series could not provide evidence of the efficacy of telerehabilitation because it was not a comparative study, our rehabilitation program was thought to have the potential to prevent the worsening of functional indices. We are conducting a prospective comparative study with a large sample size to determine the effect of telerehabilitation on functional indices.

When conducting telerehabilitation, it is extremely important to secure the safety of the participant. For this reason, we set strict inclusion criteria for this telerehabilitation program and measured the participants’ vital signs and asked participants about their condition via the telemedicine system before and after the telerehabilitation sessions. In addition, during telerehabilitation and home exercise, nursing staff members who had received lectures on rehabilitation for fall prevention observed the participants on an individual basis. As a result, no adverse events were observed during the present study.

Improvement of the cognitive status is known to be positive for the quality of life and survival of elderly people. Previous reports have shown that physical exercises had a good influence on the cognitive status of elderly people, particularly those with mild cognitive impairment.^[38,39]^ Although this study aimed to confirm the efficacy of the telerehabilitation for improving physical activity, our telerehabilitation program unexpectedly improved the MMSE scores in five participants as well as physical scales. It is still unclear why physical exercise improves the cognitive function; however, the results of the present study suggest the improvement of the cognitive function in elderly people without face-to-face rehabilitation.

The present study was associated with some limitations. First, this study employed a single-arm design. If the indices of muscle strength or body balance were improved in a large number of people who did not receive telerehabilitation, then our data would show less impact. However, it is known that almost no elderly people can recover muscle strength and body balance without rehabilitation. While the present study did not employ a comparative design, it was still significant that the quantitative scales regarding muscle strength and body balance were improved in approximately half of the participants. Second, the duration of the study was only 3 months, which might be short to assess the incidence of falls. However, the efficacy of 50 h of home-based exercise has been shown.^[40]^ In addition, although it is known that the risk of falls is closely associated with muscle strength and body balance, the present study clearly showed that our telerehabilitation could improve muscle strength and body balance, suggesting a preventive effect against falls in elderly people. Third, because only nine participants were enrolled in this study, the statistical power was limited. The characteristics of patients in whom the program was effective or ineffective are still unclear. A further analysis with a larger study population is needed to reveal the efficacy and safety of telerehabilitation for fall prevention in the elderly population.

This study illustrated that our telerehabilitation program, which included a caregiver-education program using telelectures, showed the possibility that the exercise ability of elderly participants could be improved without adverse events, and that the level of understanding regarding rehabilitation among caregivers in elderly institutions could be increased. Considering that nine participants were selected from twenty-four residents, fall prevention rehabilitation may be indicated for a large number of elderly people. We are conducting a randomized controlled study with a larger study population to validate the effectiveness of this telerehabilitation system. Telerehabilitation can be expected to play an important role in improving the physical status of elderly people, and to be beneficial for reducing the costs associated with rehabilitation.

When the rehabilitation system is applied clinically, elderly people will be able to receive high-quality rehabilitation irrespective of where they live. In addition, the system may reduce medical expenses and the burden of medical staff.

## Acknowledgments

The authors wish to thank Mr. D. Mikami for their advice on telemedicine systems.

## Author contributions

**Conceptualization:** Kentaro Moriichi, Mikihiro Fujiya, Takanori Ro, Tetsuo Ota.

**Data curation:** Kentaro Moriichi, Mikihiro Fujiya, Hitomi Nishimiya, Mariko Kodama, Nana Yoshida, Tetsuya Hosokawa, Hohei Hishiyama, Masao Kunimoto, Hiroki Hayashi, Hiroyuki Hirokawa.

**Investigation:** Takanori Ro, Tetsuo Ota, Hitomi Nishimiya, Mariko Kodama, Nana Yoshida, Tetsuya Hosokawa, Hiroki Hayashi.

**Methodology:** Kentaro Moriichi, Takanori Ro, Tetsuo Ota, Yukari Hattori.

**Project administration:** Kentaro Moriichi, Mikihiro Fujiya, Tetsuo Ota, Akitoshi Yoshida.

**Supervision:** Mikihiro Fujiya, Tetsuo Ota, Yukari Hattori, Hohei Hishiyama, Masao Kunimoto, Hiroyuki Hirokawa, Akitoshi Yoshida.

**Writing – original draft:** Kentaro Moriichi, Mikihiro Fujiya.

**Writing – review & editing:** Kentaro Moriichi, Mikihiro Fujiya, Akitoshi Yoshida.

## References

[1] Gillespie LD Robertson MC Gillespie WJ. Interventions for preventing falls in older people living in the community. Cochrane Database Syst Rev 2012;12:CD007146. 10.1002/14651858.CD007146.pub3 PMC8095069 22972103

[2] Ayed I Ghazel A Jaume-I-Capó A. Feasibility of kinectics-based games for balance rehabilitation: a case study. J Healthc Eng 2018;2018:7574860. 30123443 10.1155/2018/7574860 PMC6079427

[3] Yasumura S Hasegawa M. Incidence of falls among the elderly and preventive efforts in Japan. JMAJ 2009;52:231–6.

[4] Rubenstein LZ Jensen J. Age Ageing, 2006, Suppl 2: ii 37-ii 41. J Am Geriatr Soc 2003;51:627–35.12752837

[5] Dávalos ME French MT Burdick AE. Economic evaluation of telemedicine: review of the literature and research guidelines for benefit-cost analysis. Telemed J E Health 2009;15:933–48. 19954346 10.1089/tmj.2009.0067

[6] Dlouhý M. Measuring geographic inequalities: dealing with multiple health resources by data envelopment analysis. Front Public Health 2018;6:53. 29541631 10.3389/fpubh.2018.00053 PMC5835503

[7] Pantyley V. Health inequalities among rural and urban population of Eastern Poland in the context of sustainable development. Ann Agric Environ Med 2017;24:477–83. 28954494 10.5604/12321966.1233905

[8] Smith KB Humphreys JS Wilson MG. Addressing the health disadvantage of rural populations: how does epidemiological evidence inform rural health policies and research? Aust J Rural Health 2008;16:56–66. 18318846 10.1111/j.1440-1584.2008.00953.x

[9] Eberhardt MS Pamuk ER. The importance of place of residence: examining health in rural and nonrural areas. Am J Public Health 2004;94:1682–6. 15451731 10.2105/ajph.94.10.1682 PMC1448515

[10] Ricci-Cabello I Ruiz-Perez I Rojas-García A. Improving diabetes care in rural areas: a systematic review and meta-analysis of quality improvement interventions in OECD countries. PLoS One 2013;8 Article e84464. 10.1371/journal.pone.0084464 PMC3868600 24367662

[11] Wakerman J Humphreys JS. Sustainable primary health care services in rural and remote areas: innovation and evidence. Aust J Rural Health 2011;19:118–24. 21605224 10.1111/j.1440-1584.2010.01180.x

[12] Bradford NK Caffery LJ Smith AC. Telehealth services in rural and remote Australia: a systematic review of models of care and factors influencing success and sustainability. Rural Remote Health 2016;16:4268. 27817199

[13] Botsis T Demiris G Pedersen S. Home telecare technologies for the elderly. J Telemed Telecare 2008;14:333–7. 18852311 10.1258/jtt.2008.007002

[14] Amatya B Khan F Galea M. Rehabilitation for people with multiple sclerosis: an overview of Cochrane reviews. Cochrane Database Syst Rev 2019;1:CD012732. 30637728 10.1002/14651858.CD012732.pub2 PMC6353175

[15] Tchero H Tabue Teguo M Lannuzel A. Telerehabilitation for stroke survivors: systematic review and meta-analysis. J Med Internet Res 2018;20:e10867. 30368437 10.2196/10867 PMC6250558

[16] Dinesen B Nielsen G Andreasen JJ. Integration of rehabilitation activities into everyday life through telerehabilitation: qualitative study of cardiac patients and their partners. J Med Internet Res 2019;21:e13281. 30985284 10.2196/13281 PMC6487348

[17] Berg K Wood-Dauphine S Williams Ji. Measuring balance in the elderly: preliminary development of an instrument. Physiother Can 1989;41:304–11.

[18] Podsiadlo D Richardson S. The timed ‘Up & Go’: a test of basic functional mobility for frail elderly persons. J Am Geriatr Soc 1991;39:142–8. 1991946 10.1111/j.1532-5415.1991.tb01616.x

[19] Folstein MF Folstein SE McHugh PR. Mini-mental state. A practical method for grading the cognitive state of patients for the clinician. J Psychiatr Res 1975;12:189–98. 1202204 10.1016/0022-3956(75)90026-6

[20] Katoh M Yamasaki H. Test-retest reliability of isometric leg muscle strength measurements made using a hand-held dynamometer restrained by a belt: comparisons during and between sessions. J Phys Ther Sci 2009;21:239–43.

[21] Downs S Marquez J Chiarelli P. The Berg balance scale has high intra- and inter-rater reliability but absolute reliability varies across the scale: a systematic review. J Physiother 2013;59:93–9. 23663794 10.1016/S1836-9553(13)70161-9

[22] Lee J Geller AI Strasser DC. Analytical review: focus on fall screening assessments. PM R 2013;5:609–21. 23880047 10.1016/j.pmrj.2013.04.001

[23] Baek MJ Kim K Park YH Kim S. The validity and reliability of the mini-mental state examination-2 for detecting mild cognitive impairment and Alzheimer's disease in a Korean population. PLoS One 2016;11:e0163792. 27668883 10.1371/journal.pone.0163792 PMC5036810

[24] Palsetia D Rao GP Tiwari SC. The clock drawing test versus mini-mental status examination as a screening tool for dementia: a clinical comparison. Indian J Psychol Med 2018;40:01–10. 10.4103/IJPSYM.IJPSYM_244_17 PMC5795671 29403122

[25] Dick JP Guiloff RJ Stewart A. Mini-mental state examination in neurological patients. J Neurol Neurosurg Psychiatry 1984;47:496–9. 6736981 10.1136/jnnp.47.5.496 PMC1027826

[26] Bour A Rasquin S Boreas A. How predictive is the MMSE for cognitive performance after stroke? J Neurol 2010;257:630–7. 20361295 10.1007/s00415-009-5387-9 PMC2848722

[27] Jensen J Lundin-Olsson L Nyberg L. Fall and injury prevention in older people living in residential care facilities. A cluster randomized trial. Ann Intern Med 2002;136:733–41. 12020141 10.7326/0003-4819-136-10-200205210-00008

[28] Becker C Kron M Lindemann U. Effectiveness of a multifaceted intervention on falls in nursing home residents. J Am Geriat Sroc 2003;51:306–31. 10.1046/j.1532-5415.2003.51103.x 12588573

[29] Lamb SE Jørstad-Stein EC Hauer K. Development of a common outcome data set for fall injury prevention trials: the prevention of falls network Europe consensus. J Am Geriatr Soc 2005;53:1618–22. 16137297 10.1111/j.1532-5415.2005.53455.x

[30] Sherrington C Whitney JC Lord SR. Effective exercise for the prevention of falls: a systematic review and meta-analysis. J Am Geriatr Soc 2008;56:2234–43. 19093923 10.1111/j.1532-5415.2008.02014.x

[31] Gates S Fisher JD Cooke MW. Multifactorial assessment and targeted intervention for preventing falls and injuries among older people in community and emergency care settings: systematic review and meta-analysis. BMJ 2008;336:130. 18089892 10.1136/bmj.39412.525243.BE PMC2206297

[32] Choi YS Lawler E Boenecke CA. Developing a multi-systemic fall prevention model, incorporating the physical environment, the care process and technology: a systematic review. J Adv Nurs 2011;67:2501–24. 21545639 10.1111/j.1365-2648.2011.05672.x

[33] Howland J Hackman H Taylor A. Older adult fall prevention practices among primary care providers at accountable care organizations: a pilot study. PLoS One 2018;13:e0205279. 30307974 10.1371/journal.pone.0205279 PMC6181356

[34] Oliveira JS Sherrington C Paul SS. A combined physical activity and fall prevention intervention improved mobility-related goal attainment but not physical activity in older adults: a randomised trial. J Physiother 2019;65:16–22. 30581138 10.1016/j.jphys.2018.11.005

[35] Kovács E Prókai L Mészáros L. Adapted physical activity is beneficial on balance, functional mobility, quality of life and fall risk in community-dwelling older women: a randomized single-blinded controlled trial. Eur J Phys Rehabil Med 2013;49:301–10. 23486300

[36] Fairhall N Sherrington C Lord SR. Effect of a multifactorial, interdisciplinary intervention on risk factors for falls and fall rate in frail older people: a randomised controlled trial. Age Ageing 2014;43:616–22. 24381025 10.1093/ageing/aft204

[37] Pérula LA Varas-Fabra F Rodríguez V. Effectiveness of a multifactorial intervention program to reduce falls incidence among community-living older adults: a randomized controlled trial. Arch Phys Med Rehabil 2012;93:1677–84. 22609117 10.1016/j.apmr.2012.03.035

[38] Northey JM Cherbuin N Pumpa KL Smee DJ Rattray B. Exercise interventions for cognitive function in adults older than 50: a systematic review with meta-analysis. Br J Sports Med 2018;52:154–60. 28438770 10.1136/bjsports-2016-096587

[39] Netz Y. Is there a preferred mode of exercise for cognition enhancement in older age? –a narrative review. Front Med (Lausanne) 2019;6:57. 30984760 10.3389/fmed.2019.00057 PMC6450219

[40] Campbell AJ Robertson MC Gardner MM. Randomised controlled trial of a general practice programme of home based exercise to prevent falls in elderly women. BMJ 1997;315:1065–9. 9366737 10.1136/bmj.315.7115.1065 PMC2127698

